# Implementing Dignity-Centered Mental Health Care: Lessons from International Policy Frameworks

**DOI:** 10.3390/healthcare14070911

**Published:** 2026-04-01

**Authors:** Robert L. Anders

**Affiliations:** 1 College of Nursing, University of Texas at El Paso, El Paso, TX 79968, USA; rlanders@utep.edu; 2 Board of Trustees, Denver College of Nursing, Denver, CO 80202, USA

**Keywords:** mental health policy, human rights, dignity-centered care, nursing leadership, UNCRPD, patient safety, positive risk-taking, relational security, quality rights

## Abstract

International policy frameworks, including the United Nations Convention on the Rights of Persons with Disabilities (UNCRPD) and the WHO Quality Rights initiative, have established dignity as a foundational right in mental health care. However, a significant gap remains between these policy aspirations and the lived experience of service users, often due to risk-averse cultures that prioritize control over autonomy. This commentary employs an interpretive synthesis of international literature (2006–2025) and illustrative case examples, such as the Trieste model and Quality Rights implementation in low-resource settings, to examine the operationalization of dignity-centered care. I argue for a paradigm shift from control-based safety models to relational safety grounded in biographical literacy and positive risk-taking. Key findings highlight that dignity-centered approaches not only improve patient experiences of respect and agency but also mitigate moral injury and burnout among the nursing workforce. Furthermore, as digital mental health tools and AI-driven risk assessments emerge, systems must ensure these technologies enhance rather than automate paternalism. I conclude that realizing dignity-centered care requires a structural and cultural transformation, embedding dignity into clinical protocols, leadership practices, and environmental design to move beyond rhetorical commitments toward measurable, humane standards.

## 1. Introduction

Over the past several decades, mental health policy has undergone a significant shift from custodial, illness-centered models toward recovery-oriented and rights-based approaches. Central to this transformation is the concept of dignity. International declarations, conventions, and action plans increasingly position dignity as a foundational value guiding mental health care delivery [1,2,3,4,5]. Within these frameworks, dignity is understood not merely as an interpersonal courtesy, but as the inherent and equal worth of every human being. In mental health care, this principle carries particular significance given the historical marginalization, coercion, and systemic disempowerment experienced by persons with psychosocial disabilities [1,6].

Dignity-centered care, as used in this commentary, denotes an approach that places the inherent, equal, and non-contingent worth of every person as the organizing principle of all clinical decision-making, environmental design, and institutional policy—a standard grounded in human rights law rather than clinical effectiveness or preference satisfaction alone [7,8]. In over five decades of psychiatric nursing practice, I have observed that this term is often used interchangeably with related frameworks without sufficient precision—and that lack of clarity contributes directly to the implementation gap this commentary addresses. Dignity-centered care is distinguished from patient-centered care, which emphasizes preference satisfaction without necessarily requiring a rights-based foundation; from recovery-oriented care, which shares dignity’s emphasis on agency but is primarily clinical in scope; and from trauma-informed care, which addresses the impact of traumatic histories on engagement but does not in itself challenge coercive institutional structures. A clinician can deliver patient-centered care while still operating within a coercive institutional structure. Dignity-centered care demands more than that [1,6].

In this commentary, the terms “service users” and “patients” are used interchangeably, acknowledging ongoing debates regarding preferred terminology. When discussing international human rights instruments, the term “persons with psychosocial disabilities” is used in alignment with the United Nations Convention on the Rights of Persons with Disabilities (UNCRPD) [1].

Historical practices in institutional psychiatry continue to shape contemporary care cultures. Throughout much of the twentieth century, mental health systems were dominated by large asylums in which individuals were warehoused rather than treated, subjected to coercive practices, and excluded from decisions affecting their lives [6]. Although deinstitutionalization has reduced reliance on psychiatric hospitals in many high-income countries, paternalistic assumptions and control-oriented safety practices persist in community services, acute inpatient units, and forensic settings [9,10,11].

This commentary argues that the gap between dignity as a stated principle and dignity as lived experience remains one of the most pressing challenges in global mental health reform—a claim supported by the empirical evidence summarized in Table 1. Drawing on international policy frameworks, implementation literature, and illustrative case examples, this commentary examines how dignity is conceptualized, where implementation commonly falters, and what lessons may inform more humane and equitable systems. Rather than proposing a single model or offering definitive evaluations, the aim is to stimulate policy dialog and practice reflection across diverse contexts. I contend that dignity must move from abstract ethical aspiration to a concrete, observable, and measurable standard embedded throughout mental health systems.

While numerous publications address individual components of dignity-centered care—coercion reduction, QualityRights implementation, relational security, or digital ethics in mental health—they largely operate in isolation from one another. This commentary offers an integrative framework connecting these domains into a single coherent operational argument structured around dignity as a measurable standard. The explicit linkage between dignity-centered practice and nursing workforce moral injury, the translation of dignity principles into the operational indicators of Table 1, the empirical policy-practice gap documentation of Table 2, and the AI governance framework developed in Section 6 are each intended as additive contributions to the existing literature.

### Approach and Methodological Framing

This commentary is informed by an interpretive synthesis in the tradition of Dixon-Woods et al. (2006) [20]—a purposive, theoretically informed integration of heterogeneous literature sources rather than an exhaustive systematic retrieval. It is not a critical interpretive synthesis in the strict methodological sense and does not employ formal line-of-argument synthesis or constant comparative analysis. My search process began with the systematic identification of appropriate search terms structured around my background in health policy, psychiatric mental health nursing, and cross-cultural health systems research, including published work in the United States, Thailand, and Japan, and experience as former Dean and Professor of the College of Nursing at the University of Texas at El Paso and PI on a $6 million, five-year NIH-funded research program addressing health disparities. Searches were conducted across PubMed, PsycINFO, and Google Scholar using predefined keywords and combinations of terms relevant to the topic. Boolean operators (AND, OR) were applied to combine search terms—for example, ‘dignity AND mental health,’ ‘coercion OR restraint AND psychiatry,’ and ‘UNCRPD AND mental health implementation’—to refine results across databases and broaden or narrow the search as appropriate to the research question. Inclusion criteria encompassed peer-reviewed empirical studies, systematic reviews, implementation reports, and key international policy documents published between 2006 and 2025, with geographic diversity across high-income and low- and middle-income countries as an explicit selection criterion. The 2006 start date reflects a deliberate methodological anchor: it is the year of the UNCRPD’s adoption, which serves as the normative foundation of the entire commentary; literature predating this instrument would not reflect the rights-based policy environment under examination. Exclusion criteria excluded single case reports without broader policy relevance, opinion pieces without empirical grounding, and non-English-language sources. Titles and abstracts were screened for relevance, and duplicate and non-relevant articles were excluded. While exact database-specific retrieval counts were not systematically recorded during the initial search process, the screening process resulted in a final sample of 30 sources that met the inclusion criteria for this interpretive synthesis. While formal quality appraisal tools were not applied given the commentary format, systematic reviews and large-scale implementation studies were weighted more heavily than single-site reports. Case examples are presented illustratively rather than evaluatively and are intended to demonstrate operational approaches rather than establish causal outcomes. In terms of reproducibility, the author acknowledges that a fully detailed, database-generated record of search outputs is not available, due in part to the iterative and interpretive nature of the search process and variability in search algorithms across platforms such as Google Scholar. A formal searchable dataset of retrieved results is not available; however, the key search terms, databases, inclusion and exclusion criteria, and final source list are documented within this manuscript to support methodological transparency and allow for reasonable replication of the approach.

The author’s perspective is informed by professional backgrounds in nursing leadership, patient safety, and global health. This positioning shapes an emphasis on relational practice, workforce culture, and system-level conditions that influence the enactment of dignity in everyday care.

Case examples were selected based on their prominence in international implementation literature and their ability to represent diverse systemic challenges: the Trieste model represents a long-standing, high-resource reform success, while the Quality Rights examples illustrate the adaptability of dignity frameworks within the resource-constrained environments of low- and middle-income countries. These examples are heuristic rather than representative; successful reform cases were deliberately foregrounded to demonstrate proof of concept, and the broader evidence base includes implementation failures, partial outcomes, and contested findings that are not fully captured here.

The principal strengths of this commentary lie in the breadth of its integrative framework—connecting international policy, clinical practice, workforce culture, structural determinants, and digital ethics within a single argument—and in its translation of the normative concept of dignity into concrete operational indicators in Table 1 and the empirical evidence summary in Table 2.

## 2. International Frameworks Informing Dignity-Centered Care

International frameworks addressing dignity in mental health care operate at three distinct tiers. Legal and normative frameworks, most prominently the UNCRPD, establish dignity as an enforceable right and provide the universal normative floor. Programmatic frameworks, including the WHO Comprehensive Mental Health Action Plan and QualityRights initiative, translate legal obligations into operational guidance, assessment tools, and workforce training standards. Regional frameworks, such as those advanced by the Pan American Health Organization, contextualize these principles within specific legal systems, cultural environments, and social determinants of health. In my work across international health policy contexts, I have repeatedly observed the confusion that arises when these tiers are treated as interchangeable—they are not, and conflating them leads to unrealistic implementation expectations.

Several international policy instruments have been particularly influential in shaping contemporary understandings of dignity in mental health care. The UNCRPD represents a paradigmatic shift by asserting dignity, autonomy, and full participation as enforceable rights rather than discretionary ethical ideals [1]. Provisions addressing equal recognition before the law and liberty and security of the person directly challenge long-standing reliance on involuntary treatment and substitute decision-making [1,11].

By reframing mental distress not solely as a clinical condition but as an experience shaped by social, legal, and structural determinants, the UNCRPD mandates that health systems move beyond symptom control toward practices that affirm personhood and agency [1,6]. Recognition of legal capacity provides the normative foundation for supported decision-making, with direct implications for how risk, responsibility, and safety are conceptualized in clinical care [1,11].

The World Health Organization has translated these rights into actionable guidance through the Comprehensive Mental Health Action Plan and the QualityRights framework [2,3,4,5]. These initiatives emphasize community-based services, service user participation, and workforce development aligned with human rights standards. QualityRights, in particular, provides practical tools for service assessment, staff training, and quality improvement, enabling facilities to evaluate practices against internationally recognized benchmarks [5,18].

Regional frameworks, including those advanced by the Pan American Health Organization, further contextualize dignity within specific legal and cultural environments and explicitly link mental health to social determinants such as poverty, violence, and discrimination [21,22]. Across these instruments, common themes recur: meaningful participation of service users, accountability mechanisms, workforce development, and alignment of services with human rights standards.

While these international frameworks provide a universal legal and ethical floor, the enactment of dignity remains culturally contingent. In various global contexts, dignity may be expressed through communal belonging and family involvement rather than purely individualist notions of autonomy. Effective implementation requires a “cultural translation” of rights that respects local social structures without compromising the core principles of the UNCRPD.

## 3. Reconceptualizing Safety: From Control to Relational Practice

One of the most persistent barriers to dignity-centered care is the dominance of risk-averse cultures that equate safety with control. In many mental health settings, safety has historically been defined by the prevention of adverse events, often achieved through restrictive practices such as seclusion, restraint, and involuntary treatment [9,10,11]. This approach creates an ethical paradox, as measures intended to ensure safety may themselves cause psychological harm and undermine dignity [9,10].

Emerging scholarship challenges this paradigm by emphasizing positive risk-taking, an approach that recognizes risk as inherent to recovery and emphasizes shared decision-making [11,12]. Defensive practice, driven by fear of litigation or professional sanction, prioritizes institutional protection over therapeutic engagement. In contrast, dignity-centered safety frames risk management as a collaborative process grounded in transparency, trust, and respect for individual preferences [11,12].

Table 1 contrasts traditional control-based safety models with dignity-centered approaches, illustrating how assumptions, practices, and indicators of success shift when dignity becomes a guiding principle rather than a secondary consideration.

### 3.1. Shifting the Safety Paradigm

Reframing safety as a relational and collaborative goal shifts attention from control toward connection. Research suggests an association between highly coercive environments and elevated rates of aggression, with individuals potentially responding defensively to perceived threats to autonomy [9,10]. This relationship is observational in nature; the available evidence is heterogeneous in design, and no randomized evidence establishes direct causality. Trauma-informed perspectives further highlight how restrictive interventions can re-trigger traumatic responses, escalating rather than mitigating crises [16].

Dignity-centered safety expands the definition of a “safe outcome” beyond the absence of physical incidents to include psychological and emotional safety as reported by patients themselves [7,14]. This reframing aligns with broader patient safety movements emphasizing trust, experience, and harm prevention across multiple dimensions.

This shift is supported by evidence from coercion-reduction studies suggesting that environments which deprioritize restrictive interventions are associated with reductions in physical injuries to both staff and patients over time [9]. These findings are consistent with dignity-centered principles, though the causal contribution of the dignity framework per se remains an inference from implementation literature rather than a directly tested hypothesis. Meta-analytic conclusions are not yet supported by the available data.

### 3.2. Relational Security and the Role of Nursing

Nursing occupies a pivotal position in advancing dignity-centered care. Nurses typically spend more direct time with patients than other professionals, positioning them as key agents of relational practice [14,23]. The concept of relational security, as described in clinical guidance frameworks, suggests that staff knowledge and understanding of individual patients is associated with safer and more therapeutic care [15]. This framework reflects normative and practice-based evidence; experimental evidence for its direct causal effects on patient outcomes remains limited. In my own experience serving in senior nursing leadership at a state psychiatric hospital in Hawaiʻi—where over 50% of patients were court-ordered for treatment—the difference between staff who knew their patients and staff who did not was never theoretical. It was the difference between de-escalation and restraint.

Relational security requires biographical literacy, including awareness of personal history, trauma triggers, cultural context, and early signs of distress [15,17]. When clinicians understand agitation as a trauma response rather than willful noncompliance, interventions shift toward de-escalation, reassurance, and environmental modification, operationalizing the UNCRPD emphasis on respecting will and preferences in everyday practice [1,11].

## 4. Illustrative Case Examples from Diverse Contexts

### 4.1. The Trieste Experience as a Proof of Concept

The mental health system in Trieste, Italy, is frequently cited as an example of rights-based, community-oriented psychiatry [24]. Emerging from the deinstitutionalization movement led by Franco Basaglia, the Trieste model replaced psychiatric hospitals with a network of community mental health centers and social cooperatives grounded in principles of citizenship and social inclusion [24]. A central feature of this approach is the near-total elimination of mechanical restraint, achieved through intensive relational engagement and flexible crisis response rather than reduced safety expectations [24].

Trieste developed within a specific historical, cultural, and political context and benefits from sustained public investment and strong social services. Its relatively small catchment area and staffing ratios may limit direct replication. Nevertheless, Trieste remains valuable as a proof of concept, demonstrating that coercion is not an inevitable feature of psychiatric care when dignity and citizenship are prioritized [24].

### 4.2. Quality Rights Implementation in Low- and Middle-Income Settings

In low- and middle-income countries, implementation of dignity-centered care is often constrained by limited resources, workforce shortages, and underdeveloped mental health legislation [6,19]. WHO Quality Rights initiatives demonstrate that meaningful change is possible even within existing infrastructures. Training programs have been associated with improved staff awareness of human rights, shifts in attitudes toward coercive practices, and incremental improvements in patient experience [5,18].

Examples from Ghana, India, and Kenya illustrate how dignity principles can be adapted to local contexts while also highlighting persistent challenges, including stigma, colonial legacies of institutional care, and the need for sustained supervision in task-shifting models [6,18,19].

## 5. Workforce Development and Trauma-Informed Leadership

Embedding dignity in practice requires rethinking how mental health professionals are educated, supported, and led. Integrating human rights principles and lived experience perspectives into professional education helps bridge the gap between policy ideals and clinical realities [1,5,23].

Trauma-informed systems leadership recognizes that both service users and staff may carry trauma histories. Leaders play a crucial role in fostering environments where positive risk-taking is supported rather than punished [16,25]. Blame-oriented cultures reinforce defensive practice, whereas psychologically safe workplaces enable clinicians to prioritize relational care without fear of disproportionate sanction [25].

Furthermore, the persistence of control-oriented practices not only impacts service users, but it contributes to moral injury among the workforce. When clinicians are compelled by institutional policy to prioritize containment over therapeutic engagement, they may experience a profound sense of ethical failure. Addressing the dignity gap is therefore a prerequisite for staff wellbeing and the mitigation of burnout in high-acuity environments [13,25].

## 6. Structural Determinants and Digital Futures

Physical environments, financing mechanisms, and digital tools shape the conditions under which dignity is upheld or undermined. Evidence suggests that institutional design features such as overcrowding, lack of privacy, and poor access to nature contribute to distress and conflict, while healing environments function as preventive safety interventions [26,27].

Financing models that prioritize hospital-based care and fee-for-service reimbursement may discourage relational work and community integration [2,4,19]. Reinvestment in community-based services aligns with both human rights obligations and long-term system sustainability [2,4].

The COVID-19 pandemic exposed and accelerated pre-existing dignity deficits in mental health systems globally. Community-based services that had never been adequately resourced faced significant disruption under pandemic conditions. Relational care—the foundation of dignity-centered practice—was disrupted by isolation protocols and visitor restrictions at precisely the moment patients were most vulnerable. Reports from some settings suggested increases in involuntary admissions and heightened reliance on control-oriented responses as systems faced systemic stress, though the evidence base remains uneven across contexts. As someone who taught and practiced through the pandemic, I observed firsthand how quickly dignity gains can be surrendered when institutions face systemic pressure. This experience reinforces the central argument of this commentary: dignity-centered care must be structurally embedded rather than dependent on individual practitioner goodwill.

Digital mental health technologies present new opportunities and risks. While telehealth and AI-assisted tools may expand access, they also raise concerns regarding surveillance, algorithmic bias, data privacy, and depersonalization [28,29,30,31]. A dignity-centered digital framework requires that technology enhance rather than replace human relationships, with safeguards for informed consent, transparency, and accountability [28,29]. Specifically, the rise in algorithmic risk-prediction tools threatens to automate the “source of risk” view identified in Table 1. If AI models are trained on historical data from coercive systems, they risk codifying past paternalism into future care. A dignity-centered digital approach must ensure that algorithms do not replace biographical literacy with static risk scores that ignore a patient’s evolving agency and context.

Illustrative examples of AI tools currently deployed or under development in mental health settings include natural language processing systems applied to clinical notes for suicide risk stratification, and predictive readmission models used in acute inpatient settings. Veterans, court-ordered patients, and individuals from marginalized communities are precisely the groups most likely to be harmed by algorithmically encoded bias—the populations this commentary is most concerned with protecting. Documented concerns include racial and socioeconomic bias in training data, lack of transparency in algorithmic decision-making, and absence of mechanisms for patient challenge or override [30,31]. A minimum ethical governance standard for dignity-compatible digital mental health practice requires: algorithmic transparency and bias auditing prior to deployment; meaningful informed consent processes that do not treat AI-assisted assessment as a standard clinical default; clear accountability pathways when algorithmic outputs contribute to restrictive decisions; and ongoing alignment review against UNCRPD dignity standards [1,31].

## 7. Measuring Progress Without Reducing Dignity to Metrics

Concrete dignity indicators can support accountability and quality improvement, including autonomy support, reduction in coercive practices, integration of peer support, informed consent fidelity, and patient-reported experiences of respect and agency [7,8,9,11,14]. These measures are most effective when used as learning tools rather than punitive benchmarks.

Resource constraints, data burden, and risks of performative compliance must be acknowledged, particularly in low-resource settings. Dignity indicators should guide reflective improvement cycles rather than function as isolated performance targets [7,8]. It is important to note that the call for measurable standards in this commentary is aspirational. Available measurement instruments are heterogeneous in design and validation, cross-cultural applicability remains limited, and no single standardized tool has achieved international consensus [7,8]. Having worked across clinical and policy settings in multiple countries—including environments as different as a forensic psychiatric hospital in Hawaiʻi and community health systems in Southeast Asia—I am acutely aware that ‘measurable dignity’ means very different things across contexts. This commentary’s advocacy for measurable dignity standards is therefore intended to stimulate instrument development and validation research, not to imply that such tools are presently available at scale. The contribution of this commentary on the measurement question is to name the gap and frame the research agenda—not to claim the gap has been closed.

## 8. Implications and Future Directions

Advancing dignity-centered mental health care requires coordinated action across policy, organizational, clinical, and community levels. Sustained progress depends on resisting reactive responses to isolated incidents and investing in relational, trauma-informed, and community-based solutions [11,16].

Specific research priorities include: (1) longitudinal cohort studies examining the sustained impact of dignity-indicator frameworks on patient experience outcomes across diverse healthcare system types; (2) cost-effectiveness analyses comparing relational-security-based models with traditional containment-based approaches in acute inpatient settings; (3) cross-cultural validation studies for patient-reported dignity experience measures, particularly in LMIC contexts where existing instruments lack normative data; and (4) ethics review frameworks specifically designed to evaluate AI-assisted risk prediction tools against UNCRPD dignity standards prior to clinical deployment [1,7,19,30,31].

## 9. Limitations

Several limitations of this commentary warrant explicit acknowledgment. First, the evidence base is heterogeneous, spanning normative policy documents, qualitative implementation studies, systematic reviews of variable methodological quality, and clinical guidance frameworks; claims have been calibrated to the level of evidence available, but direct comparisons across study types carry inherent limitations. Second, the case examples selected—Trieste and WHO QualityRights implementations—are prominent reform successes and do not fully represent the range of international implementation experiences; selection bias toward successful cases is an acknowledged feature of the purposive sampling approach. Third, the available English-language literature on dignity implementation may reflect publication bias, with positive reform experiences more likely to reach indexed sources than negative or mixed outcomes, particularly from LMIC settings. Fourth, the relationship between coercive environments and patient aggression discussed in this commentary is associational rather than causal; reverse causality—whereby patient aggression may precipitate coercive responses—cannot be excluded from observational evidence. Fifth, the framing of dignity as individual autonomy, which reflects the UNCRPD’s foundational architecture, may not fully capture collective or relational understandings of dignity operative in non-Western contexts. These limitations are inherent to the commentary format and are offered to assist readers in appropriately contextualizing the arguments presented.

## 10. Conclusions

International policy frameworks articulate a compelling vision for dignity-centered mental health care, but realizing this vision requires sustained implementation across system levels. Dignity must be embedded in clinical protocols, leadership practices, environmental design, and financing structures. Evidence from diverse contexts demonstrates that alternatives to coercive psychiatry are achievable [5,18,24]. The remaining question is whether systems are willing to undertake the relational, cultural, and structural transformation required to make dignity a lived reality rather than a rhetorical commitment.

## Figures and Tables

**Table 1:** Comparison of traditional safety models and dignity-centered safety models in mental health care [9,10,11,12,13]. Rows in blue reflect additions made in response to reviewer feedback.

Feature	Traditional Safety Model	Dignity-Centered Safety Model
Primary goal	Eliminate clinical risk	Promote autonomy and personhood
View of the patient	Source of risk to be managed	Partner with lived experience and agency
Core approach	Risk aversion and control	Positive risk-taking and collaboration [11,12]
Typical practices	Seclusion, restraint, involuntary treatment [9,10]	De-escalation, supported choice, advance planning [11]
What “safety” emphasizes	Absence of incidents	Psychological, emotional, and physical safety as experienced by the patient [14]
Key metrics	Counts of incidents and rule compliance	Coercion reduction and patient-reported respect/autonomy [7,8,9]
Staff training focus	Risk assessment and containment protocols	Trauma-informed communication, relational security, de-escalation [12,15,16]
Success indicator	“Zero incidents” culture	Therapeutic alliance quality and recovery progression [14,17]
Governance structures	Hierarchical, clinician-led accountability; incident-driven review	Multi-stakeholder, rights-based accountability; service user participation in governance [1,5]
Decision-making processes	Clinician-directed; substitute decision-making	Shared decision-making; advance planning; supported decision-making [1,11]
Role of service users	Passive recipient of care decisions	Active participant; co-design of care; peer support integration [5,18]
Evaluation indicators	Incident counts; compliance rates; staff safety metrics	Coercion reduction rates; patient-reported experience measures; peer support utilization [7,8,9]

**Table 2:** Selected empirical evidence on the policy-practice gap in dignity-centered mental health care.

Author(s), Year	Setting	Study Type	Key Finding Related to Dignity Gap
Drew et al., 2011 [6]	Global	Policy review	Documents widespread human rights violations against persons with mental and psychosocial disabilities persisting despite international rights commitments.
Patel et al., 2018 [19]	Global/LMIC	Lancet Commission report	Landmark Lancet Commission on global mental health and sustainable development addressing equity, access, and structural determinants of mental health globally; among the barriers identified are resource constraints, stigma, and inadequate rights-based legislation as systemic impediments to equitable care in low- and middle-income countries [19].
Gooding et al., 2020 [11]	International	Scoping review	Finds coercive practices remain prevalent globally despite policy commitments to reduction; risk-averse institutional cultures identified as a key implementation barrier.
Barbui et al., 2021 [9]	International	Systematic review	Umbrella review of randomized evidence finding that staff training, shared decision-making, and integrated care interventions show benefit for reducing coercive treatment; community treatment orders and adherence therapy showed no significant effect. Calls for further research in high- and low-income countries to strengthen the evidence base [9].
Chieze et al., 2019 [10]	Adult psychiatry	Systematic review	Documents psychological and physical harms of seclusion and restraint, highlighting the ethical paradox whereby safety measures may themselves violate dignity.
Funk & Drew, 2017 [18]	Global/WHO	Policy commentary	Policy commentary describing the WHO QualityRights initiative and its global momentum in transforming attitudes and practices toward human rights alignment in mental health services; frames QualityRights as a primary vehicle for implementing CRPD obligations in clinical and community settings [18].

## Data Availability

Data sharing is not applicable to this article as no new datasets were generated or analyzed during the current study.
